# Editorial: Advances in Using Big Data and Artificial Intelligence to Understand Heterogeneity in Inflammatory Responses

**DOI:** 10.3389/fimmu.2022.948885

**Published:** 2022-06-22

**Authors:** Xu-jie Zhou, Amanda S. MacLeod, Lam Cheung Tsoi

**Affiliations:** ^1^ Renal Division, Peking University First Hospital; Kidney Genetic Center, Peking University Institute of Nephrology; Key Laboratory of Renal Disease, Ministry of Health of China, Beijing, China; ^2^ Department of Dermatology; Molecular Genetics and Microbiology, Duke University, Durham, NC, United States; ^3^ Department of Immunology; Molecular Genetics and Microbiology, Duke University, Durham, NC, United States; ^4^ Janssen Research and Development, Spring House, PA, United States; ^5^ Department of Dermatology, University of Michigan, Ann Arbor, MI, United States; ^6^ Department of Computational Medicine & Bioinformatics, University of Michigan, Ann Arbor, MI, United States; ^7^ Department of Biostatistics, University of Michigan, Ann Arbor, MI, United States

**Keywords:** artificial intelligence, big data, heterogeneity, immune-mediated disorders, multi-omics, precision medicine

In this Research Topic, we have hosted 3 in-depth reviews and 15 original research articles presenting how novel technological, methodological, and conceptual advancements can be integrated to study the underlying mechanisms that drive the heterogeneity in inflammatory responses among patients suffering from immune-mediated conditions.

The immune system plays a vital role in health and disease, and is regulated through a complex interactive network of immune cells and mediators, thus multi-omics approach in immunological research is advocated to provide a better understanding of the system. As biomedical research transitioning into data-rich science, an era of “big data” emerged owing to these advancements. The integration of such multi- layered datasets with longitudinal assessments of patient outcomes has the capacity to shed important lights into different aspects of disease pathogenesis, progression and cell-specific responses, with which to guide design of targeted therapies. Multi-source big data is thus suggested to be the major driver of precision medicine. However, only data alone can be hardly to be transformed into clinically actionable knowledge, if we don’t have proper analysis methods. Thanks to the advances of computing science, artificial intelligence (AI) is developed for robust data analysis.

Chu et al. extensively introduced multi-omics approaches in immunological research, and Orrù et al. reviewed that systematic multi-parametric flow cytometry coupled with high-resolution genetics and transcriptomics can be used to reveal endophenotypes of autoimmune diseases for therapeutic development. Through AI-based analysis of different disease parameters – including clinical and para-clinical outcomes, and molecular profilesl from multi-omic data, a digital twin paired to the patient’s characteristic can be created, enabling healthcare professionals to handle large amounts of patient data, and Voigt et al. discussed the use of digital twins for MS as a revolutionary tool to improve diagnosis, monitoring and therapy refining. Digital twins will help make precision medicine and patient-centered care a reality in everyday life. At the level of genomics, through genome-wide association study (GWAS), Connell et al. found a genome-wide significant association between intergenic variant rs35569429 and response to ustekinumab for the treatment of moderate to severe psoriasis. These work also discuss how AI and multi-omics can be applied and integrated, to offer opportunities to develop novel diagnostic and therapeutic means in immune related diseases.

Using transcriptomic analysis on skin biopsies, Abernathy-Close et al. observed that skin-associated B cell responses distinguish discoid lupus erythematosus (DLE) from subacute cutaneous lupus erythematosus (SCLE) and acute cutaneous lupus erythematosus (ACLE). This data has important implications for trial design for patients with isolated cutaneous lupus erythematosus (CLE). Maruyama et al. conduced RNA-seq data analysis and identified several lncRNAs such as *MALAT1, CA3-AS1, GASAL1, PSMA3-AS1, MIR4435-2HG, IL21-AS1, AC111000.4*, and *LINC01501*, and some of them are associated with active *Visceral leishmaniasis* infection. By implementing the weighted gene co-expression network analysis (WGCNA), Zhang et al. suggested that the osteoarticular involvement in psoriasis and ankylosing spondylitis (AS) could be mediated by the mRNA surveillance pathway. Also based on RNA-Seq expression, Cao et al. observed that *RIMKLB* expression is associated with survival outcomes and tumor-infiltrating immune cells (TIICs) in patients with colorectal cancer (CRC), indicating that it might be a potential novel prognostic biomarker that reflects the immune infiltration status. There are also different studies in our Research Topics that utilize single cell genomics approaches. Xu et al. performed single-cell RNA sequencing, demonstrating cell-specific transcriptional profiles in the kidney, anti- phospholipase A2 receptor (*PLA2R*) positive membranous nephropathy (MN) -associated novel genes, signaling pathways involved, and potential pathogenesis concerning ligand-receptor interactions. Liu et al. took single-cell RNA-sequencing of CD45+cells isolated from active lesions of patients with psoriasis vulgaris, they found *CXCL13* significantly correlated with the severity of lesions and genes elevated in psoriatic skin-resident memory T cells are enriched for programs orchestrating chromatin and CDC42-dependent cytoskeleton remodeling. Alber et al. used single cell CITE-Seq (Cellular Indexing of Transcriptomes and Epitopes by sequencing) technology to analyze peripheral blood mononuclear cells (PBMCs) in ankylosing spondylitis (AS) and identified a number of molecular features which were associated with AS were linked with inflammation and other immune-mediated diseases. With the increasing resources of single-cell sequencing data, issue of heterogeneity and limited comprehension of chronic autoimmune disease pathophysiology could be better addressed. Ma et al. integrated several sets of single-cell RNA sequencing data and bulk RNA-sequencing data from open access database deposited in the Gene Expression Omnibus (GEO), and found that the interactions among the peripheral blood mononuclear cells (PBMCs) subpopulations of SLE patients may be weakened under the inflammatory microenvironment. With transcriptomic datasets in ulcerative colitis, Chen et al. applied artificial neural network (ANN) and identified a predictive RNA model in which combination of *CDX2, CHP2, HSD11B2, RANK, NOX4*, and *VDR* was a good predictor of patients’ response to infliximab (IFX) therapy. Liu et al. performed single cell profiling of transcriptome and cell surface protein expression to compare the peripheral blood immunocyte populations of individuals with psoriatic arthritis (PSA), individuals with cutaneous psoriasis (PSO) alone, and healthy individuals. They observed a higher abundance of Tregs and dnT cells in PSA patients and a higher abundance of hematopoietic stem precursor cells (HSPCs) in healthy subjects.

O’Neil1 et al. sought to identify serum proteomic alterations that dictate clinically important features of stable rheumatoid arthritis, and couple broad-based proteomics with machine learning to predict future flare. They defined 4 proteomic clusters reflecting biological mechanisms, and found an XGboost machine learning algorithm could classify patients who relapsed with an AUC of 0.80 using only baseline serum proteomics. We can also see that some novel data or perspectives were discussed and shared from different groups. For example, Cao et al. took two-sample bidirectional Mendelian randomization analysis and cross-trait meta-analysis between major depressive disorder (MDD) and atopic diseases (AD: asthma, hay fever, and eczema). They found a significant genetic correlation between MDD and ADs, and detected a major causal effect of genetic liability to depression on ADs. Jamerson et al. explored the heterogeneity of atopic dermatitis and psoriasis between African American and European American patients by summarizing epidemiological studies, addressing potential molecular and environmental factors, with a focus on psychosocial or psychological stress on immune pathways, and highlighted the role of the hypothalamus-pituitary-adrenal (HPA) axis and *IL-18* in atopic dermatitis, corticotropin-releasing hormone and brain derived neurotrophic factor in psoriasis, and cortisol levels in both. It supports environmental components in disease heterogeneity and their influence on disease pathogenesis. Observational studies may also shed some light on precision medicine. By retrospectively reviewing the Taiwan National Health Insurance Research Database (NHIRD) within 13 years, Li et al. observed that influenza vaccination was associated with lower asthma risk in patients with AD.

The use of AI and multi-omics in human diseases are still in their infancy, mostly for research. Although it is premature to try and define the potential clinical utility of these newly found molecule biomarkers or predictive models, they do provide an important impetus for further studies that aim to further define a biological definition of sub-phenotypes in patients with immune related diseases that can ultimately guide clinical decision making (**Figure 1**).

**Figure 1:**
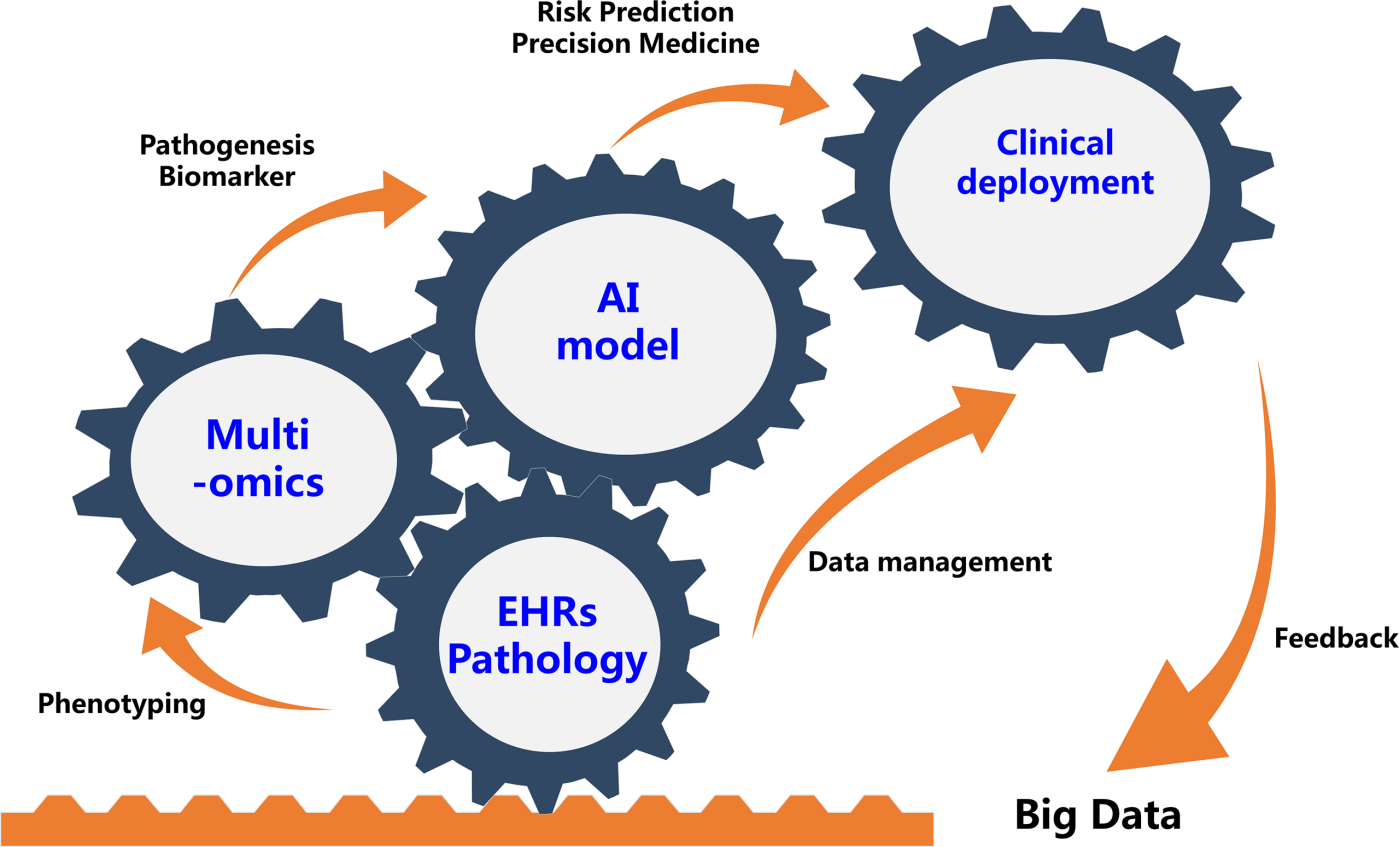
Future Integration of Artificial Intelligence And Multi-omics Will Benefit Precision Medicine for Immune-Mediated Disorders. EHRs, electronic health records.

## Author Contributions

All authors listed have made a substantial, direct and intellectual contribution to the work, and approved it for publication.

## Funding

This work was supported by Beijing Natural Science Foundation (Z190023).

## Conflict of Interest

The authors declare that the research was conducted in the absence of any commercial or financial relationships that could be construed as a potential conflict of interest.

## Publisher’s Note

All claims expressed in this article are solely those of the authors and do not necessarily represent those of their affiliated organizations, or those of the publisher, the editors and the reviewers. Any product that may be evaluated in this article, or claim that may be made by its manufacturer, is not guaranteed or endorsed by the publisher.

